# Palindromic Sequence-Targeted (PST) PCR, Version 2: An Advanced Method for High-Throughput Targeted Gene Characterization and Transposon Display

**DOI:** 10.3389/fpls.2021.691940

**Published:** 2021-06-22

**Authors:** Ruslan Kalendar, Alexandr V. Shustov, Alan H. Schulman

**Affiliations:** ^1^National Laboratory Astana, Nazarbayev University, Nur-Sultan, Kazakhstan; ^2^Viikki Plant Science Centre, HiLIFE Institute of Biotechnology, University of Helsinki, Helsinki, Finland; ^3^National Center for Biotechnology, Nur-Sultan, Kazakhstan; ^4^Natural Resources Institute Finland (Luke), Helsinki, Finland

**Keywords:** genome walking, transposon display (TD), palindrome, restriction site, amplified fragment length polymorphism (AFLP), transposable elements (TE), biodiversity

## Abstract

Genome walking (GW), a strategy for capturing previously unsequenced DNA fragments that are in proximity to a known sequence tag, is currently predominantly based on PCR. Recently developed PCR-based methods allow for combining of sequence-specific primers with designed capturing primers capable of annealing to unknown DNA targets, thereby offering the rapidity and effectiveness of PCR. This study presents a methodological improvement to the previously described GW technique known as palindromic sequence-targeted PCR (PST-PCR). Like PST-PCR, this new method (called PST-PCR v.2) relies on targeting of capturing primers to palindromic sequences arbitrarily present in natural DNA templates. PST-PCR v.2 consists of two rounds of PCR. The first round uses a combination of one sequence-specific primer with one capturing (PST) primer. The second round uses a combination of a single (preferred) or two universal primers; one anneals to a 5′ tail attached to the sequence-specific primer and the other anneals to a different 5′ tail attached to the PST primer. The key advantage of PST-PCR v.2 is the convenience of using a single universal primer with invariable sequences in GW processes involving various templates. The entire procedure takes approximately 2–3 h to produce the amplified PCR fragment, which contains a portion of a template flanked by the sequence-specific and capturing primers. PST-PCR v.2 is highly suitable for simultaneous work with multiple samples. For this reason, PST-PCR v.2 can be applied beyond the classical task of GW for studies in population genetics, in which PST-PCR v.2 is a preferred alternative to amplified fragment length polymorphism (AFLP) or next-generation sequencing. Furthermore, the conditions for PST-PCR v.2 are easier to optimize, as only one sequence-specific primer is used. This reduces non-specific random amplified polymorphic DNA (RAPD)-like amplification and formation of non-templated amplification. Importantly, akin to the previous version, PST-PCR v.2 is not sensitive to template DNA sequence complexity or quality. This study illustrates the utility of PST-PCR v.2 for transposon display (TD), which is a method to characterize inter- or intra-specific variability related to transposon integration sites. The *Ac* transposon sequence in the maize (*Zea mays*) genome was used as a sequence tag during the TD procedure to characterize the *Ac* integration sites.

## Introduction

Targeted genomic regions for which the nucleotide sequences are not known can be captured for sequencing and other applications by a variety of methods collectively known as genome walking. Based initially on the creation of libraries of cloned DNA fragments and screening for individual clones, GW approaches have evolved considerably to become rapid and efficient protocols that are independent of cloning. The introduction of PCR in the early 1990s radically changed GW approaches, as GW is currently predominantly based on PCR. Modern PCR-based methods allowed GW on template DNA from human, animal, plant, fungal, bacterial, and viral origins.

A common requirement for PCR utilization in GW is the presence in a targeted template DNA of a region (which may be quite short) for which the nucleotide sequence is known or at least may be predicted. This region is used as a “sequence tag” to bind a sequence-specific primer (SSP) or a set of “nested” SSPs. In most methods (Shyamala and Ferro-Luzzi Ames, 1993; Grivet et al., 2001; Rishi et al., 2004; Tan et al., 2005; Wang et al., 2007, 2011; Leoni et al., 2008; Reddy et al., 2008; Tonooka and Fujishima, 2009; Tsuchiya et al., 2009; Bae and Sohn, 2010; Ji and Braam, 2010; Liu et al., 2013; Trinh et al., 2014; Chang et al., 2018; Schrader and Schmitz, 2018; Li et al., 2019; Ashrafmansouri et al., 2020; Zeng et al., 2020; Fraiture et al., 2021), nested SSPs and multiple rounds of amplification are used to improve the specificity of GW. SSPs may target a range of homologous sequences, making the same set of SSPs suitable for GW with a series of related species or genes. During PCR amplifications, a common companion to an SSP is a “walking” primer that anneals to the template having an unknown sequence. Accordingly, the sequence of the walking primer frequently incorporates modifications, such as sequence degeneracy, to allow arbitrary annealing.

Established PCR-based methods include ligation-dependent PCR, randomly primed PCR, and thermal asymmetric interlaced (TAIL) PCR. For example, ligation-dependent PCR relies on digesting of a template DNA with a restriction enzyme (RE), ligating restriction fragments to a short synthetic adaptor, and performing PCR with an SSP and a primer targeting the adaptor’s sequence. Randomly primed PCR uses multiple successive rounds of amplification; published procedures include three nested SSPs and four degenerate primers (DPs). The SSPs anneal to a known sequence tag and have high melting temperatures (T_m_). In contrast, the DPs are sufficiently degenerate to anneal to nearly random sites in natural DNA templates; the calculated T_m_ of the DPs are correspondingly low. As an improvement to randomly primed PCR, TAIL-PCR was developed, which has the distinctive feature of an elaborate thermal cycling profile that relies on decreasing binding efficiency of the DPs at high annealing temperatures (Kalendar et al., 2019). A modification to the TAIL-PCR exists, which is deemed to provide higher efficiency (hiTAIL-PCR) (Liu and Chen, 2007). The latter procedure combines the advantages of TAIL-PCR and suppression-PCR (Rand et al., 2005), and was reported to have improved efficiency.

All PCR-based methods for GW are potentially sensitive to off-target annealing of walking primers and concomitant generation of non-specific products [dubbed random amplified polymorphic DNA (RAPD)-like products]. For particular methods, optimization strategies were published to provide the best balance between sensitivity and specificity. Nevertheless, sufficiently enriching for PCR products that contain a GW-capture target is not always possible. For example, two “traditional” methods (inverse PCR and TAIL-PCR) suffer from significant amplification of non-specific PCR products despite optimization. Amplification is also sensitive to template DNA quality. In this regard, ligation-dependent PCR is extremely sensitive, and requires isolation of high-quality DNA followed by digestion with a methylation-insensitive RE and ligation with adapter(s) before PCR amplification. This multi-stage process is laborious and frequently inefficient. The generation of RAPD-like products remains a problem. In fact, on average, side products constitute the majority of amplification results for all the aforementioned methods. Based on our experience, if three or more PCR rounds with different SSPs or walking primer(s) must be performed, this always indicates either that the method lacks insufficient sensitivity or specificity, or that the primers or template require optimization.

At present, increasing utilization of next-generation sequencing (NGS) appears to be an alternative that in theory could make GW obsolete. However, NGS requires access to expensive equipment and personnel trained in bioinformatics; thus, NGS is not justified for all situations. Moreover, current NGS relies on sufficient amounts of high-quality template DNA, which is not always available. The high costs and labor associated with library preparation for NGS make it suitable for comparative analysis of only a limited number of samples. When the task may have an unlimited number of samples, such as for genetically modified organism (GMO) testing in foods, the routine use of NGS is unjustified and redundant. NGS-produced results are highly dependent on the accuracy of the sequence assembly from raw reads, leading to the necessity of experimental confirmation of sequences in cases of high responsibility. It is likely that GW utilization will persist for the experimental confirmation of sequences flanking a known sequence tag. Moreover, methods originally developed for GW appeared to be convenient tools for tasks that are not “traditional” GW, such as population genetics studies or identifying GMO events or adulteration of foods. Nevertheless, NGS data can provide sequence tags for GW, thus linking two major methodologies of genomic research.

Use of GW for population genomics is increasing, as exemplified by a growing number of publications. The reasons include improved methods that are more efficient in terms of sensitivity and specificity, combined with reduced labor costs, compared with previously used GW methods. GW combined with NGS as a source of sequence tags solves potential ambiguities in NGS results, providing increased accuracy of genomic fragment characterization. This combination appears optimal in terms of costs for characterizing genomic segments of different but related organisms or in component genomes coexisting in polyploid organisms.

We have previously proposed a method called palindromic sequence-targeted PCR (PST-PCR) (Kalendar et al., 2019), which is rapid (requires only two PCR rounds), allows easy control of the balance between sensitivity and specificity, and is superior in performance to other published methods. The distinctive features of PST-PCR include walking primers (PST primers) that anneal to a template, not randomly but arbitrarily, and a two-phase thermal profile. Walking primers for PST-PCR anneal to short (6-bp long) palindromic sequences (PST sites) that have theoretically predictable locations in the target DNA. Such annealing is not random, as the sequence of the PST site itself is defined. The molecular structure of a PST primer is sequentially as follows: a defined (non-degenerate) sequence complementary to a PST site at the 3′-terminus; a degenerate sequence of sufficient length (6–12 nt); a universal sequence (adaptor region) at the 5′-terminus. In the previously proposed PST-PCR process (Kalendar et al., 2019), one PST primer partners with one SSP during the first round, whereas in the second round the same SSP is used in a combination with a universal (adaptor) primer. The adaptor primer anneals to the adaptor sequence present at the 5′-terminus of the PST primer.

The thermal profile during PST-PCR includes a phase of linear amplification (driven by an SSP only) followed by an exponential amplification phase during which both the SSP and PST primers participate in PCR. Importantly, the T_m_ is sufficiently high in both phases to prevent annealing to sequences other than PST sites. The result is the specific amplification of a GW product. The efficient annealing of PST primers to PST sites even at high T_m_ in conjunction with site-specific annealing of SSPs makes the PST-PCR process appear and perform much like a conventional PCR on a simple template; only one or two PCR rounds (30–36 cycles in a total) are needed to produce a PST-PCR product in significant amounts.

In this report, we describe an improved approach to the previously published version (Kalendar et al., 2019), here called version 2 (PST-PCR v.2). Similar to PST-PCR, in PST-PCR v.2 the first-round amplification uses one SSP paired with a PST primer. The difference between PST-PCR v.2 and its predecessor is that the second round uses single-primer amplification with just one primer (adaptor primer) that is unrelated to a targeted template. To use just one universal primer for re-amplification, both the SSP and PST-PCR v.2 PST primer carry an identical adaptor sequence at their 5′-termini. PST-PCR v.2 is even faster than its predecessor and simpler to design and optimize. Moreover, to our surprise, we discovered that the use of single-primer amplification in the second round further reduces the generation of non-specific (RAPD-like) amplicons and the formation of primer oligomers. The PST-PCR v.2 protocol is suitable for routine high-throughput amplification of unknown DNA fragments, transposon display (TD), and the capture of homologs to known genes in other species. The PST-PCR v.2 method preserves attractive features of the original PST-PCR, as this new version is rapid, taking no more than 2–3 h, and is unaffected by template sequence complexity or input DNA quality.

Transposon display is a variation of ligation-dependent PCR. TD involves digesting genomic DNA with a RE, ligating restriction fragments with an adaptor, and performing PCR. The traditional approach uses a paired SSP-adaptor primer; the SSP targets a sequence tag within the transposon. As the flanking RE sites are also different for insertions of the transposon into different sites in genomic DNA, TD provides information on the variability of the insertions in a particular genome, which can be visually presented in the form of amplification products of various sizes separated in an electrophoretic gel. Moreover, TD is suitable for finding variation among individuals if the level of polymorphism of insertional sites is sufficient.

For almost any genome diversity study in which the original protocol includes RE digestions and ligations, PST-PCR v.2 is a more feasible alternative. In this study, we demonstrated the utility of PST-PCR v.2 by employing it in a TD task. SSPs targeting sequences of the *Ac* transposon in the maize (*Zea mays*) genome in combination with PST primers were used to produce GW fragments representing polymorphisms of *Ac* insertional sites in several parental and hybrid lines. We show that PST-PCR v.2 is an attractive alternative to existing strategies to study insertion-site variability even in complex plant genomes.

## Materials and Methods

### Plant Material and DNA Extraction

Grains of maize lines and hybrids were kindly provided by the Agricultural Research Center (ARC) and the United States Department of Agriculture (USDA). As stated by the USDA, “this germplasm is being freely distributed by the United States National Plant Germplasm System (NPGS) educational, agricultural research, or breeding purposes.” We used them only for agricultural research purposes. Furthermore, none of the germplasm was indicated by the USDA to be patented or legally protected. Note that on the shipping list, none are listed as restricted. Leaves for DNA isolation were collected from 12-day-old plants. Genomic DNA was extracted using a CTAB-based protocol and treated with RNase A (doi: 10.17504/protocols.io.mghc3t6) (Kalendar et al., 2021a). DNA samples were diluted in 1X TE buffer (10 mM Tris–HCl pH 7.5, 1 mM EDTA) and DNA quality was verified using a Nanodrop spectrophotometer (Thermo Fisher Scientific) and gel electrophoresis.

### PST Primer Design

Palindromic sequence-targeted primers were designed to meet the following conditions: a 6-nt palindromic sequence is present at the 3′ end; a 6–10 nt fully degenerate sequence (dN_6__–__10_) is present upstream (i.e., in the direction of the 5′-terminus) of the palindrome; an adaptor region with a distinctive sequence is present upstream of the degenerate sequence. The invariable adaptor in this study was 19 nt (Table 1). The calculated T_m_ and GC content for the PST primers are presented in Table 1. The T_*m*_ were computed for the 16-nt “core” sequences (in this study we define the palindrome together with the degenerate sequence as core sequence), as only the core sequences of the PST primers are expected to anneal to template DNA. T_m_ was calculated using a nearest-neighbor thermodynamic model with the following reaction conditions: 50 mM monovalent cation (K^+^/NH_4_^+^), 2 mM Mg^2+^, and 0.5 μM of PST primer. Thermodynamic calculations and modeling of primer secondary structures were performed using FastPCR software (Kalendar et al., 2017a, b, c). Linguistic complexity (LC), a formal measure of informational content in a nucleotide sequence, was computed for palindromic sequences. As an important practical note, we recommend screening PST primers for any task. For example, in an experiment we recommend setting up multiple PST-PCR amplifications that have the same template and SSP, together with different PST primers from Table 1.

**TABLE 1:** Cycling conditions for PST-PCR v.2.

**Reaction type**	**Number of cycles**	**Thermal conditions**
**First round**		
		95°C (2 min)
Linear	18–20	95°C (15 s), 72°C (1 min)
Exponential	1–3	95°C (15 s), 55–65°C (30 s), 72°C (1 min)
**Second round**		
		95°C (1 min)
Exponential	28	95°C (15 s), 72°C (60 s)

### Target-Specific Primer Design

As an example for target-specific design, sequences of *Ac* transposons from *Z. mays* were downloaded from GenBank. Sequences of individual *Ac* transposons were extracted and used to build multiple alignments. Regions within both termini that were highly conserved in all *Ac* transposons were used to target the SSPs. Sets of *Ac*-specific primers were designed to target the termini of the selected transposon. The SSPs are shown in Table 3. The following rules were used for selection of the SSPs: each SSP should be 25–35 nt, have a GC content of 40–60%, and a calculated T_m_ ≥ 65°C. A good SSP should not be capable of forming self-dimers. Generally, the *Ac*-specific primers should be as close to the end of the known sequence as possible. The SSPs were designed using FastPCR software with the following calculation parameters: 50 mM monovalent cation (K^+^/NH_4_^+^), no divalent cations, and a working primer concentration of 0.25 μM.

**TABLE 2:** Primers for PST-PCR.

**ID**	**Sequence (5′-3′)**	**Restriction site**	**Tm (°C)**	**CG (%)**	**LC (%)**
**Tail primer (for second round PCR)**					
5600	GTTGCGGCAGGTCCTCACC	–	69.1	68.1	89
**PST primers (for first round PCR)**					
5601	GTTGCGGCAGGTCCTCACCnnnnnnnnnnGACGTC	*Aat*II	49.3	56.3	100
5602	GTTGCGGCAGGTCCTCACCnnnnnnnnnnAACGTT	*Acl*I	46.1	43.8	100
5603	GTTGCGGCAGGTCCTCACCnnnnnnnnnnTTCGAA	*Ass*II	45.6	43.8	100
5604	GTTGCGGCAGGTCCTCACCnnnnnnnnnnTGGCCA	*Bal*I	51.0	56.3	100
5605	GTTGCGGCAGGTCCTCACCnnnnnnnnnnGGATCC	*Bam*HI	48.3	56.3	100
5606	GTTGCGGCAGGTCCTCACCnnnnnnnnnnTGATCA	*Bcl*I	44.7	43.8	100
5326	GTTGCGGCAGGTCCTCACCnnnnnnnnnnAGATCT	*Bgl*II	43.7	43.8	100
5607	GTTGCGGCAGGTCCTCACCnnnnnnnnnnATCGAT	*Cla*I	44.8	43.8	89
5608	GTTGCGGCAGGTCCTCACCnnnnnnnnnnGAATTC	*Eco*RI	43.7	43.8	100
5609	GTTGCGGCAGGTCCTCACCnnnnnnnnnnGATATC	*Eco*RV	42.3	43.8	89
5610	GTTGCGGCAGGTCCTCACCnnnnnnnnnnAAGCTT	*Hin*dIII	45.5	43.8	100
5611	GTTGCGGCAGGTCCTCACCnnnnnnnnnnGTTAAC	*Hpa*I	43.6	43.8	100
5612	GTTGCGGCAGGTCCTCACCnnnnnnnnnnGGTACC	*Kpn*I	48.2	56.3	100
5613	GTTGCGGCAGGTCCTCACCnnnnnnnnnnCCATGG	*Nco*I	49.1	56.3	100
5614	GTTGCGGCAGGTCCTCACCnnnnnnnnnnGCTAGC	*Nhe*I	49.3	56.3	89
5615	GTTGCGGCAGGTCCTCACCnnnnnnnnnnCACGTG	*Pma*CI	50.2	56.3	100
5616	GTTGCGGCAGGTCCTCACCnnnnnnnnnnCTGCAG	*Pst*I	49.7	56.3	100
5617	GTTGCGGCAGGTCCTCACCnnnnnnnnnnCAGCTG	*Pvu*II	49.7	56.3	100
5618	GTTGCGGCAGGTCCTCACCnnnnnnnnnnGAGCTC	*Sac*I	48.8	56.3	100
5619	GTTGCGGCAGGTCCTCACCnnnnnnnnnnGTCGAC	*Sal*I	49.3	56.3	100
5620	GTTGCGGCAGGTCCTCACCnnnnnnnnnnAGTACT	*Sca*I	43.7	43.8	100
5621	GTTGCGGCAGGTCCTCACCnnnnnnnnnnGCATGC	*Sph*I	50.9	56.3	89
5622	GTTGCGGCAGGTCCTCACCnnnnnnnnnnAGGCCT	*Stu*I	50.1	56.3	100
5327	GTTGCGGCAGGTCCTCACCnnnnnnnnnnTCTAGA	*Xba*I	43.1	43.8	100
5623	GTTGCGGCAGGTCCTCACCnnnnnnnnnnCTCGAG	*Xho*I	48.6	56.3	100

*T_*m*_, melting temperature; GC content calculated for the core sequence (3′-end palindrome and 10 degenerate positions) at primer concentration of 0.5 μM in the presence of 2 mM Mg^2+^; for the tail primer, calculations were made for 0.25 μM primer in absence of Mg^2+^; LC (%), linguistic complexity calculated for 6-nt restriction sites.*

**TABLE 3:** Sequence-specific primers (SSPs) for the *Ac* transposon from *Zea mays.*

**ID**	**Sequence (5′-3′)**	**Information (KM013689)**	**T_m_ (°C)**	**CG (%)**	**LC (%)**
5633	GTTGCGGCAGGTCCTCACCcggtgaaacggtcgggaaactagctct	154←180	63.9	55.6	78
5634	GTTGCGGCAGGTCCTCACCcgtccgatttcggctttaacccgacc	4038→4067	64.0	57.7	73
5635	GTTGCGGCAGGTCCTCACCagatgtagcaagtggctcctctccatgagc	18814→18843	64.5	53.3	83

*T_*m*_, melting temperature calculated for a primer concentration of 0.25 μM in the absence of Mg^2+^; CG% percentage of C and G; LC (%), linguistic complexity.*

### PST-PCR Setup and Parameters

Palindromic sequence-targeted PCR was performed as a two-round PCR. In the first round, various combinations of the walking primer (PST) with the target-specific (SSP) primer were used. Reaction mixtures were prepared for which each particular reaction contained one SSP (for every SSP from Table 3) and one PST primer (for every PST primer listed in Table 1). All first-round amplifications were performed using the same reaction conditions. Upon completion of the first round, the first-round reaction mixtures were added as templates to the second-round reaction mixtures. The primer in the second-round reactions was single universal tail primer (5600 in Table 1). The thermal cycling parameters for the two rounds of the PST-PCR process are shown in Table 2.

### Example Protocol for First-Round PCR

The first-round PCR was performed in a 30-μL reaction mixture consisting of 30 ng template DNA, 1x *Taq* reaction buffer, and 1 U Taq DNA Polymerase (NEB). The reaction contained 2 mM Mg^2+^, 200 μM each dNTP, 0.2 μM SSP, and 0.5 μM PST primer. The first round used the following thermal profile: Initial denaturation (95°C for 2 min); 20 cycles of linear amplification (95°C for 15 s and 72°C for 60 s); 1–5 cycles of exponential amplification (95°C for 15 s, 55–65°C for 30 s, and 72°C for 60 s. The final extension step was at 72°C for 2 min. The reaction mixture was used as a template for the second-round amplification.

### Example Protocol for Second-Round PCR

The reaction mixtures (30 μl) consisted of 1x *Taq* reaction buffer, 1 U OneTaq DNA Polymerase (NEB), and 0.4 μM tail primer (5600). A total of 1 μl of the product from the first round (not diluted) was added to the reaction mixtures described above. The thermal cycling profile was 95°C for 1 min (initial denaturation), then 28 two-step cycles (95°C for 15 s and 70°C for 90 s), and a final extension at 72°C for 2 min. The PCR products were separated by electrophoresis at 70–90V for 3 h in a 1.2% agarose gel (Wide Range, SERVA Electrophoresis GmbH) in 0.5X TBE electrophoresis buffer. Gels were stained with EtBr and scanned using an FLA-5100 imaging system (Fuji Photo Film GmbH) at a resolution of 50 μm. Selected PCR products were cloned for sequencing.

### PST-PCR v.2 for Transposon Display

Maize inbred lines (A619, A632, B73, and Mo17) and their hybrids (A619 × A632 and B73 × Mo17) were studied. The first-round PCR was performed in 40 μL of a reaction mixture consisting of 40 ng template DNA, 1 × OneTaq reaction buffer, 1 U OneTaq DNA Polymerase, 200 μM each dNTP, 0.2 μM SSP, and 0.3 (Figure 3) to 0.5 μM (Figure 2) PST primer. During the first round, a linear amplification was performed as follows: 95°C, 2 min (initial denaturation) followed 22 cycles of 95°C for 15 s and 72°C for 90 s. The first round was then continued with an exponential amplification for 3 cycles (95°C for 15 s, 60°C for 30 s, and 72°C for 60 s). For the second round, 2 μl of the product was added to 38 μl of a reaction mixture composed of 1 × OneTaq reaction buffer and 1 U OneTaq DNA Polymerase (NEB), 200 μM each dNTP, and 0.4 μM tail primer (5600). The thermal cycling profile was as follows: 95°C for 1 min (initial denaturation) and 23 (Figure 2) or 29 (Figure 3) two-step cycles (95°C for 15 s and 70°C for 60 s). Products of the second-round PCR were analyzed by gel electrophoresis. Product sizes were determined against GeneRuler DNA Ladder Mix 100–10,000 bp (Thermo Fisher Scientific). Gels were stained with EtBr and scanned with a FLA-5100 imaging system (Fuji Photo Film GmbH) at a resolution of 50 μm.

**FIGURE 1:**
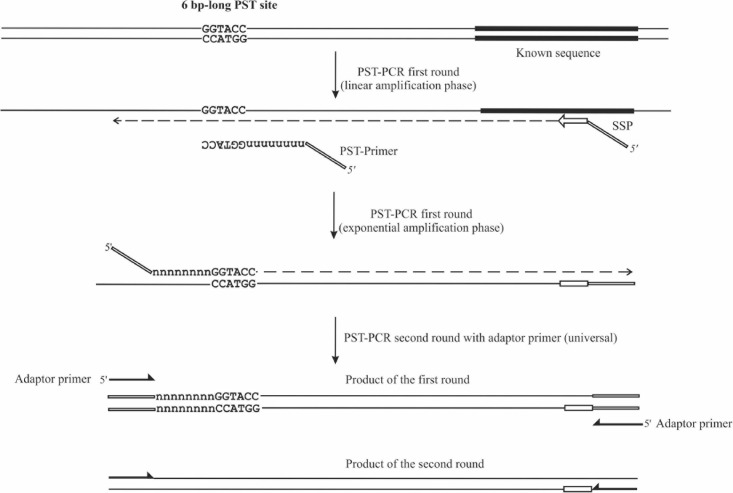
Schematic depiction of PST-PCR v.2. The figure shows two consecutive PCR rounds. The first round was performed with a 5′-tailed sequence-specific primer (SSP) and one PST primer. The SSP primer anneals to a target with a known sequence. The PST primer anchors to a palindromic sequence in a region with an unknown sequence. The second round was performed with a single-tail primer. The regions with known sequences are depicted with thick lines. Other regions of the template with unknown sequences are shown as thin lines.

**FIGURE 2:**
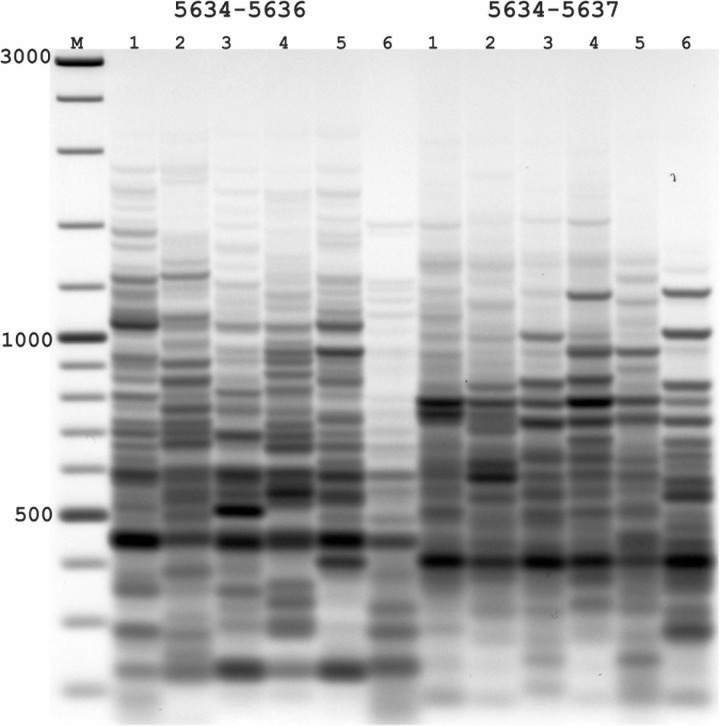
Results of applying PST-PCR v.2 as a transposon display (TD) for *Ac* elements in the maize genome. Lanes represent samples of maize lines and hybrids: 1,7, A632 (PI587140); 2,8, hybrid A619 × A632 (Ames23710); 3,9, A619 (PI587139); 4,10, Mo17 (PI558532); 5,11, hybrid B73 × Mo17 (Ames19097); 6,12, B73 (PI550473). Lane M – size markers. In the first round, SSP primer 5634 (Table 2) was paired with one of the PST primers (1–6: 5634-5636; 7–12: 5634-5637, Table 2). Primer 5600 (Table 2) was used in the second round. Results of the second-round PCR detected *Ac* elements TD polymorphisms.

**FIGURE 3:**
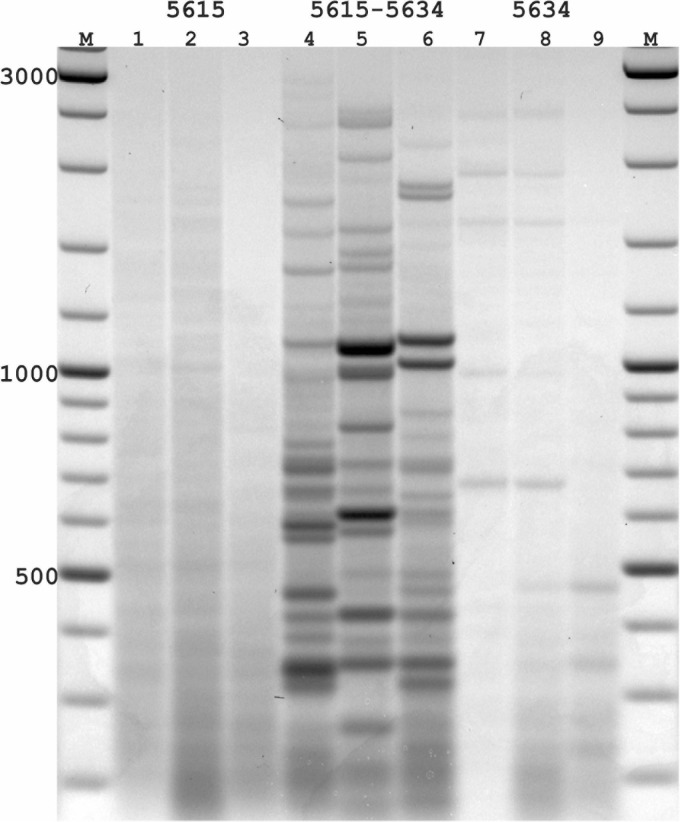
PST-PCR v.2 used for transposon display (TD) for *Ac* elements in the maize genome. Lanes represent PST-PCR v.2 amplification results for maize lines and a hybrid: 1, A632 (PI587140); 2, hybrid A619 × A632 (Ames23710); 3, A619 (PI587139). Lane M – size markers. Only one primer (PST primer 5615) was present during the first-round PCR for samples in lanes 1–3. Both primers (SSP 5634 and PST primer 5615) were present during the first-round PCR for samples in lanes 4–6. Only one primer (SSP 5634) was present during the first-round PCR for samples in lanes 7–9. A specific and efficient amplification is visible only when both SSP and a PST primer are present in the first round.

## Results

### PST-PCR Version 2 Principles, Starting Protocol, and Possibility of Customization

The PST-PCR v.2 process (schematically depicted in Figure 1) is based on a specific design for walking primers (PST primers). At the 3′ end, a PST primer has a defined sequence that is 6 (or sometimes 8 nt long) and is palindromic (i.e., resembles a type II RE site). The palindrome is followed by a degenerate sequence (6–12 nt long). We refer to the palindrome together with the degenerate sequence as the primer “core”. The core sequence is capable of annealing by base-pairing to 12–18 nt long contiguous stretches of nucleotides in a targeted template. During the process of selecting PST primers, the primer’s T_m_ is computed for the core sequence. Importantly, the annealing of the primer’s 3′ end is possible only to the palindrome in the template. The targeted palindrome is called a PST site. Finally, a universal (invariable) sequence is present at the PST primer’s 5′-terminus, which is called the adaptor region. The adaptor region is 19 nt long in the PST primers (Table 1), however, the length and nucleotide sequence of the adaptor region is at the discretion of the user.

An important characteristic of the process contributing to PST-PCR v.2 performance is the thermal profile of first-round amplification. The thermal profile of the first round consists of two phases (Table 2). At PCR start with a linear amplification phase, only an SSP anneals to the template and serves to generate single-stranded DNA. The linear amplification phase is followed by an exponential amplification phase, during which both primers (SSP and PST primer) anneal to respective targeted sites and drive the exponential accumulation of an amplification product. As the SSP’s T_m_ is higher than that of the PST primer, the amplification is switched from the linear to exponential phase by decreasing the annealing temperature. As shown previously (Kalendar et al., 2019), the efficiency of the accumulation of ssDNA templates (SSP-primed) during the linear phase determines the overall specificity of PST-PCR, whereas efficiency of the accumulation of a PCR product (with both SSP and PST primer) defines final yields and the sensitivity of the process. PST-PCR efficiency is dependent on T_m_ at both amplification phases and from a critical cycle number at which the specificity-favoring T_m_ is changed to sensitivity-favoring T_m_. While any software can be used to calculate the optimal T_m_, the process of optimization of PST-PCR for a particular target is reduced to empirically determining the optimal critical cycle number. Therefore, the process is easily but finely controlled by choosing the critical cycle number. It is recommended (Kalendar et al., 2019) to perform 12–20 cycles (first round) at a high T_m_ (70–72°C) and additionally 5–7 cycles at a reduced T_m_ (55–65°C).

Although the PST-PCR process in some cases results in sufficiently amplifying a PCR product during a single (first) amplification round, in most cases one more round is needed to produce more product. Accordingly, in the originally described PST-PCR second round, an SSP is paired with a universal adaptor primer (i.e., the primer targeting the adaptor region at the 5′-terminus of a PST primer). To expedite the process, the thermal cycling profile during the second round includes two steps (annealing and extension are combined in one step). The improved method presented herein is different in that only the second-round PCR uses a single-primer amplification with only the adaptor primer. To target the adaptor primer at both ends of a PCR product, the newly designed SSP carries the same adaptor region at the 5′-terminus as in the PST primer. This methodological modification significantly simplifies the development of working protocols for various tasks, including those distant from classical GW.

Byproducts of primer-dimers are occasionally generated in PCR due to inadvertent annealing of one primer on another and subsequent extension of such hybrids with Taq polymerase. If present in a reaction mixture, primer-dimers consume primers and compete with the synthesis of the desired product. The authors empirically found that using the single-primer second round diminishes the formation of primer-dimers, presumably by reducing the chances of forming primer hybrids.

It is recommended that during preliminary studies, a set of different PST primers (as many as feasible, but no less than four) is tested in combinations with any SSP(s) and a novel (previously uncharacterized) template (Kalendar et al., 2019). We observed that screening of various PST primers increases the probability of success, as testing a sufficient number of combinations (various PST primers plus one SSP) universally leads to amplifying the desired product. For example, all PST primers listed in Table 1 might be tested in a particular study design. However, upon completion of the initial screening, it may be possible to utilize only the selected primer pairs for a routine characterization of related templates. The performance of different PST primers also varies in processes involving different templates. However, the general recommendation is that the best PST primers have a 6 nt long 3′-terminal palindrome, and the best core sequences have 67–100% GC content.

Careful attention should be paid to SSP selection. The optimal SSP is 26–32 nt long and has a 40–65% GC content (Kalendar et al., 2011, 2017a). We also recommend that the last 12 bases of the 3′ end of the hybridizing part of the primer Tm’s should preferably not exceed 42°C, and the last 12 bases of the 5′ end of the hybridizing part of the primer Tm’s should preferably be at least 42°C. In primer-design software, the long-distance PCR option should be chosen (Kalendar et al., 2011, 2017a). The working concentration of the SSP is 0.2–0.3 μM; the SSP is used in a two-step PCR (combined annealing/extension) with annealing temperature 68–72°C. The choice of DNA polymerase is also important for PST-PCR v.2. Preferred DNA polymerases lack proofreading (3′→5′ exonuclease) activity. This is presumably because the 3′→5′ exonuclease destroys the 3′-end of the PST primer, leading to possible random annealing (of the primer) outside of PST sites and reducing specificity.

### Transposon Display for the *Ac* Element Family

In this study, the *Ac* transposable element was selected as a model to show the efficiency of the PST-PCR v.2 approach in a TD-based genetic diversity study. The TD was chosen because the efficiency of this method can be conveniently demonstrated in an example of genetic inheritance of the *Ac* elements in several parental and hybrid lines of maize. The genetic properties of Activator (*Ac*) and Dissociation (*Ds*) “controlling elements” were discovered by Barbara McClintock when she was studying maize cytogenetics; these were later identified as DNA transposons (Fedoroff et al., 1983). The *Ac* element is autonomous, whereas *Ds* requires the presence of *Ac* to transpose. Through transposition, both *Ac* and *Ds* can insert into functional genes, causing mutations that alter phenotype, which however, may revert fully or partially to wild type if the inserted element excises. The 4.6-kb autonomous *Ac* element encodes a single protein, the transposase, and is bounded by 11-bp terminal inverted repeats (TIRs). The element contains multiple copies of a hexameric repeat within the terminal 200 bp of both ends, to which the *Ac* transposase binds (Du et al., 2011). The *Ac* and *Ds* insertions create 8-bp target site duplications (TSD).

In the absence of transpositional activity, *Ac* and *Ds* insertions will be inherited vertically. The more genetically distant two parental lines are from each other, the greater the differences in their *Ac* insertion sites and the consequent amplification products and pattern in the TD. Likewise, PCR bands present in the parental lines must be also present in a hybrid line (Figure 2). For maize lines whose genome has been completely sequenced (e.g., assembly Zm-B73-REFERENCE-NAM-5.0), all genomic *Ac* integration sites in the voucher sample are known. The available sequence information allows interpretation of the appearance of individual bands in the TD PCR and validation of the apparent transposon dynamics observed by TD.

The nested SSPs targeting the termini of the *Ac* sequence are listed in Table 3. These SSPs were paired with PST primers (Table 1) to test all possible combinations (outer SSP and PST primer). PCR products of various lengths were produced in PST-PCR v.2 with high yields and no empiric optimization beyond the recommended experimental conditions. One example of an electrophoretic gel with the separated PCR products is shown in Figure 2. All PST-PCR v.2-generated DNA fragments were isolated from gels, cloned, and sequenced. As a demonstration of the method’s performance, all bands appeared to be desired GW products representing amplified *Ac* insertion sites.

While varying PCR conditions and studying their influence on generating specific vs. non-specific PCR-products from the maize templates, we tested several DNA-polymerase brands; varied numbers of PCR-cycles for linear and exponential amplification stages; and tested particular conditions of SSP or PST primers in generating non-specific products in the resulting PCR mixtures. One representative experiment is presented in Figure 3. In this experiment, the possibility of generating non-specific (RAPD-like) PCR products was tested in conditions when only one primer (either SSP or a PST primer) was present in the first round. The second round was performed with a single adaptor primer as intended in the PST-PCR v.2 protocol. In Figure 3, samples for parental maize lines and hybrid lines produced efficient and specific amplification only when both the SSP and a PST primer were present in the reaction mixture during the first round. In contrast, if only SSP or a PST primer alone were used for the first round, and a different (adaptor) primer was used for the second round, the resulting products contained only smears of low intensity (barely visible by the naked eye; detected using a gel-imaging device) and no major bands were visible in any sample. Moreover, reducing the number of PCR cycles at the first round reduced even the smear appearance, in addition to preventing the non-specific bands.

With PST-PCR v.2, reaction conditions can be found at which discrete PCR products are produced only for a combination of SSP + PST primer [as is exemplified by the pair SSP (5634) + PST (5615), Figure 3]. We recommend performing such control experiments during initial testing of PST-PCR v.2 with new templates. The same procedure tests for the specificity of SSP primers. The selection of SSPs is also of paramount importance for entire method. With DNA templates that carry several SSP-annealing regions in proximity and in inverted orientations (as in the case of closely spaced mobile elements), a single SSP can anneal to such templates, which leads to the generation of PCR amplicons that resemble products of the Inter-Repeat Amplification Polymorphism (IRAP) method (Kalendar and Schulman, 2006; Kalendar et al., 2021b).

For PST primers, the possibility of generating RAPD-like amplicons also depends on the reaction conditions, specifically the parameters of exponential-phase amplification when a PST primer participates in the process. In this regard, if the number of PCR cycles during the exponential phase (in the first round) increases past certain threshold (∼20 cycles), it is theoretically possible to generate RAPD-like products representing template fragments that are flanked by two PST sites (specific to a used PST primer) and positioned within a distance of efficient amplification (up to 3,000 bases). Thus, the exponential phase of the first round should not be performed with a large number of cycles (one to three cycles are enough in most cases; we recommend doing no more than five cycles at this stage).

However, the ability of PST primers to participate in RAPD-like amplification can be used on its own if a sufficient number of PCR cycles is allowed, particularly when PST-PCR is used for DNA fingerprinting. In the latter case, PST-PCR is a preferred alternative to ligation-dependent PCR approaches, such as amplified fragment length polymorphism (AFLP) (Vos et al., 1995). The authors are currently investigating the utility of using such PST-PCR and a manuscript is under development.

## Discussion

Genotyping has become routine in controlling production, transportation, and consumption of plants and plant-based produce. We present a novel method for capturing unsequenced DNA fragments from whole-genome templates as an alternative to other genotyping methods (Liu and Whittier, 1995; Tan et al., 2005; Leoni et al., 2008; Bae and Sohn, 2010; Wang et al., 2011). We call this PST-PCR v.2, a GW technique based on a distinctive design of walking primers and SSPs. PST-PCR v.2 is applicable to whole-genome amplification as an alternative to AFLP, sequence-specific amplified polymorphism (SSAP), multiple banding pattern techniques (Waugh et al., 1997), and methods to identify GMO transgene insertion flanks (Liu and Chen, 2007) as part of the regulatory regimes. For this purpose, SSPs may be targeted to known conserved transgenic events or the T-DNA sequence (Liu and Chen, 2007). The method requires the presence of only a small sequence tag for which the nucleotide sequence is known. In comparison to the originally described PST-PCR, the defining feature of PST-PCR v.2 is the use of a single primer PCR at the reamplification stage of the process. PST-PCR v.2 preserves the high sensitivity and specificity of PST-PCR; however, PST-PCR v.2 is even more rapid and amenable to utilization for tasks beyond traditional GW. Such utilization was demonstrated during *Ac* TD with the maize genome.

### Optimization

Several technical issues are important for the success of PST-PCR. A properly selected SSP is of paramount importance; SSP selection guidelines are presented here. Although PST-PCR v.2 is specific in generating the targeted products, as for any GW method, one should not remain alert to off-target amplifications. Further improvement of the specificity is possible by using nested SSPs. A second but nevertheless important consideration is that multiple different PST primers must be tested in preliminary experiments. We provide a list of 30 PST primers for which the ability to drive the PST-PCR process has been shown. After selecting the SSP and PST primers, other optimization aspects to be considered include annealing temperature during the first-round amplification and the threshold cycle number at which the annealing temperature is switched. In case dropouts (missing amplicons for known targets) are suspected, decreasing the linear-phase annealing temperature is recommended.

Template DNA may naturally contain sites matching particular PST primers within an interval amplifiable by Taq polymerase. In this case, PST-PCR may suffer from undesirable capturing and amplification of such fragments. Although regions flanked by PST sites are expected to occur in natural templates, the inadvertent amplification of such fragments is surprisingly not a persistent problem in PST-PCR. The authors sequenced dozens of PST-PCR products and did not find one flanked at both ends with a PST primer. It is likely that a product containing an SSP target at one end and a PST target at the other has a competitive advantage during amplification over PST-PST side products, as the SSP anneals faster than the PST primer. This is because the PST primer is highly degenerate (and thus a reaction mixture) and the effective concentration of annealing-capable variants for the PST primer is lower than the SSP concentration.

### Use of PST-PCR v.2 Beyond Genome Walking

The method presented herein has broad utility in population studies, including biodiversity monitoring, genotyping, and pedigree confirmation. For such applications it is possible to target SSPs at mobile genetic elements, such as long interspersed nuclear elements (LINEs), short interspersed nuclear elements (SINEs), LTR retrotransposons, and DNA transposons (Kalendar and Schulman, 2014). Mobile genetic elements are multi-copy targets and large parts of their sequences are evolutionarily conserved, thus simplifying the selection of SSPs.

As a demonstration of the utility of PST-PCR v.2 beyond the single-copy targets of GW, we used it to display insertional polymorphisms of the *Ac* transposon. The high levels of polymorphism observed suggest that the *Ac* transposon has been active since the divergence of various maize genetic lineages. The method was able to track individual transpositional events in genetic lines and to characterize hybrids. It appears that PST-PCR v.2 is suitable for studying the population dynamics of transposon families in either selfing or out-crossing species. Virtually all *Ac* insertions identified in an inbred maize line were present in all individual representatives from this line. We also observed unique bands among the individual samples from inbred lines used in this study.

Although differences in element abundance between different lines were pronounced, there was an approximately similar number of insertion sites per individual in maize. However, given the sequence analysis of a significant fraction of the identified insertion sites, the small sizes of the regions amplified, and the overall small number of insertion sites, the variability in banding pattern was likely due to insertional polymorphism. The method herein allows simultaneous amplification of many individual insertion sites. As described, the method allows confirmation of the assumption that the variability in *Ac* insertion sites results mainly from insertional events that occurred within the line, rather than from hybridization of different lines. Furthermore, the amplification products generated by this method allow for sequencing to provide molecular confirmation of the genetic hypotheses.

PST-PCR v.2 does not require using nested SSPs, unlike the first version, thus preserving the specificity of the original PST-PCR. Moreover, PST-PCR v.2 may be multiplexed, utilizing combinations of several SSPs plus PST primers that have different 5′-terminal adaptor sequences. In this case, two or more pairs of SSP and PST primers are present in a single first-round PCR; both primers in one pair carry the same adaptor sequence and primers in different pairs differ in the adaptor sequences. Correspondingly, a second-round PCR will include two or more different adaptor primers. This approach could be used in population studies targeting multicopy genes or transposable elements, particularly to provide indexes to save costs in library construction for subsequent NGS.

In this study, PST-PCR v.2 is presented as a novel genome walking and fingerprinting method that may be used widely due to its significant information capacity, low cost, and minimal requirements for optimization on a particular template. PST-PCR v.2 may be used in initial descriptions of intraspecific and interspecies genetic variability and to track lines and genotypes. The authors are currently developing a high-throughput fingerprinting platform for plant genomes using the protocols presented herein.

## Data Availability Statement

The raw data supporting the conclusions of this article will be made available by the authors, without undue reservation.

## Author Contributions

RK conceptualized the project, developed the methods and software, performed the experiments, analyzed the data, and prepared the figures. RK, AVS and AHS wrote the manuscript. RK and AHS acquired funds for the project. All authors contributed to the article and approved the submitted version.

## Conflict of Interest

The authors declare that the research was conducted in the absence of any commercial or financial relationships that could be construed as a potential conflict of interest.
